# ID4-dependent secretion of VEGFA enhances the invasion capability of breast cancer cells and activates YAP/TAZ via integrin β3-VEGFR2 interaction

**DOI:** 10.1038/s41419-024-06491-2

**Published:** 2024-02-06

**Authors:** Anna Benedetti, Chiara Turco, Enzo Gallo, Theodora Daralioti, Andrea Sacconi, Claudio Pulito, Sara Donzelli, Claudia Tito, Martina Dragonetti, Letizia Perracchio, Giovanni Blandino, Francesco Fazi, Giulia Fontemaggi

**Affiliations:** 1 Translational Oncology Research Unit, IRCCS Regina Elena National Cancer Institute, Rome, Italy; 2 Department of Pathology, IRCCS Regina Elena National Cancer Institute, Rome, Italy; 3 Biostatistics and Bioinformatics Unit, Clinical Trial Center, IRCCS Regina Elena National Cancer Institute, Rome, Italy; 4 Department of Anatomical, Histological, Forensic & Orthopaedic Sciences, Section of Histology & Medical Embryology, Sapienza University of Rome, Rome, Italy

**Keywords:** Non-coding RNAs, Breast cancer

## Abstract

Understanding the mechanisms of breast cancer cell communication underlying cell spreading and metastasis formation is fundamental for developing new therapies. ID4 is a proto-oncogene overexpressed in the basal-like subtype of triple-negative breast cancer (TNBC), where it promotes angiogenesis, cancer stem cells, and BRACA1 misfunction. Here, we show that ID4 expression in BC cells correlates with the activation of motility pathways and promotes the production of VEGFA, which stimulates the interaction of VEGFR2 and integrin β3 in a paracrine fashion. This interaction induces the downstream focal adhesion pathway favoring migration, invasion, and stress fiber formation. Furthermore, ID4/ VEGFA/ VEGFR2/ integrin β3 signaling stimulates the nuclear translocation and activation of the Hippo pathway member’s YAP and TAZ, two critical executors for cancer initiation and progression. Our study provides new insights into the oncogenic roles of ID4 in tumor cell migration and YAP/TAZ pathway activation, suggesting VEGFA/ VEGFR2/ integrin β3 axis as a potential target for BC treatment.

## Introduction

Cell migration and invasion are finely regulated processes critical in normal and physiological conditions, including embryonic development, wound repair, and immune surveillance [1–3]. However, dynamic cell movements are also crucial in cancer progression and metastasis [4]. Over 90% of cancer mortality is due to metastatic tumor growth. Therefore, understanding the mechanisms by which cells migrate and invade may help to predict and prevent lethal metastatic disease [5].

Focal adhesions (FAs) are macromolecular protein structures at the plasma membrane that connect the intracellular actin cytoskeleton with the Extracellular Matrix (ECM), providing a mechanical link with the extracellular environment [6–8]. On the ECM side, transmembrane integrin receptors bind ECM components such as collagen and fibronectin and become activated by clustering. On the cytoplasmic side, integrins bind paxillin, vinculin, FAK, talin, tensin, and hundreds of other proteins that link the FA complex to the actin filaments, stimulating the formation of actin stress fibers [9, 10]. Among the FA proteins, Focal Adhesion Kinase (FAK) is activated in different cancer types, including breast cancer, where it promotes tumor progression [11]. In cancer, the activation of tyrosine kinase receptors stimulates tumor cell migration and invasion by interacting with integrins and promoting FA assembly and turnover [11]. Integrin clustering induces FAK activation by autophosphorylation on Tyr 397, which promotes a conformational change exposing the binding sites for other proteins belonging to the Src family. These proteins start a phosphorylation cascade activating downstream targets, leading to tumor cell migration, growth, and promoting cancer stem cells [11–13].

Integrins are crucial regulators of cell mechanotransduction: by sensing ECM stiffness they coordinate cell tensional state response. FAK activity triggered by integrin clustering stimulates the activation of the Hippo pathway members’ YAP and TAZ, two important mediators of cell mechanotransduction [14, 15]. YAP and TAZ are retained in the cytoplasm by LATS1/2 proteins when inactive, but upon FA activation, SRC activates YAP/TAZ and inhibits LATS1/2 [15]. Once in the nucleus, YAP and TAZ bind the DNA through TEAD transcription factors and induce the transcription of target genes involved in tumor cell survival, metastasis formation, cancer stem cell promotion, drug resistance, and tumor metabolism [16]. Understanding the molecular mechanisms leading to YAP/TAZ activation is necessary to develop targeted therapies that may induce tumor cell differentiation and drive the cancer to a less aggressive phenotype. YAP/TAZ pathway could also be targeted by interfering with the tumor ECM stiffness or with other components of the tumor microenvironment, such as immune cells or cancer-associated fibroblasts [16, 17].

ID4 (inhibitor of differentiation 4) is a dominant negative regulator of the basic helix-loop-helix transcription factors associated with breast cancer aggressiveness, particularly with the basal-like subtype of triple-negative breast cancer (TNBC), where it associates with reduced survival and acts as an independent prognostic factor [18]. In addition, ID4 controls various properties of TNBC, including the stem cell potential, the angiogenic potential, and DNA repair activity [19–21].

We have previously reported that ID4 controls BC angiogenesis by inducing the expression of CXCL1, IL-8, and VEGFA. In particular, VEGFA production by BC cells induces the differentiation of tumor-associated macrophages toward a pro-angiogenic phenotype [22, 23]. However, the role of ID4 in BC cell migration and invasion is unknown. This study shows that ID4-mediated VEGFA production activates focal adhesion signaling in BC cells by stimulating integrin β3 and VEGFR2 interaction, promoting cell migration and invasion. Moreover, the activation of FAs induced by VEGFA promotes YAP/TAZ nuclear translocation and pathway activation.

## Results

### ID4 expression correlates with motility-related pathways in breast cancer

**Fig. 1:**
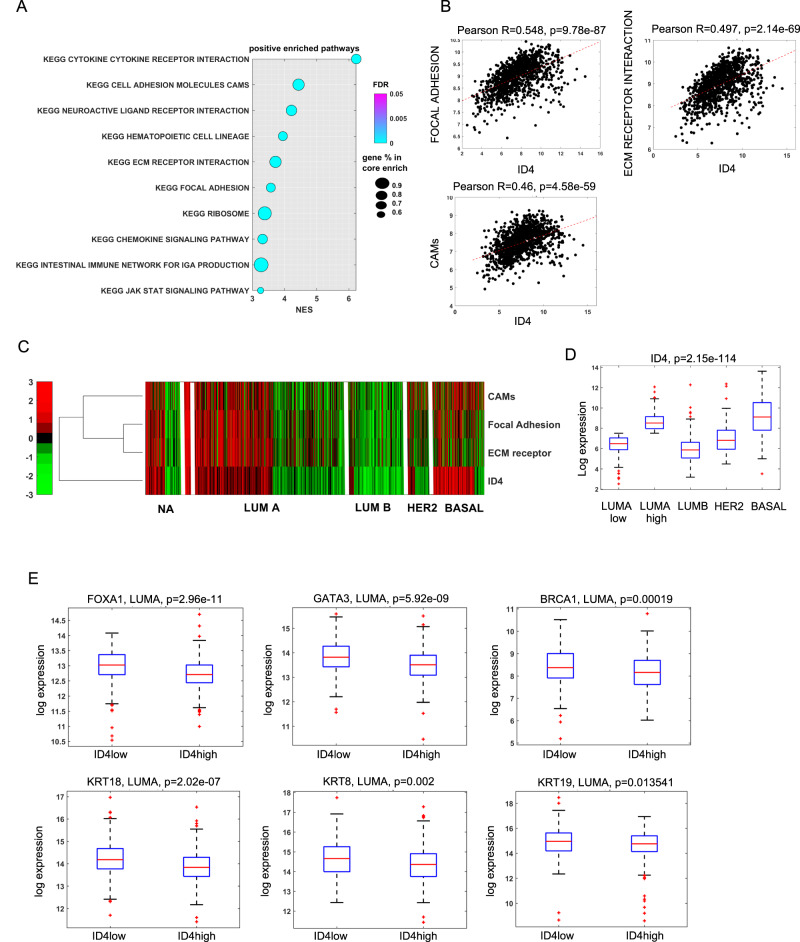
ID4 expression correlates with motility-related pathways in breast cancer. **A** gene sets enrichment analysis showing activated pathways in BC expressing high vs. low levels of ID4. **B** graphs showing the correlation between ID4 expression and focal adhesion pathway, ID4 expression and ECM receptor interaction pathway, and ID4 expression and CAM KEGG pathway in breast cancer patients. **C** heatmap derived from TCGA BRCA dataset showing ID4 expression and the activation of CAM pathway, Focal adhesion pathway, and ECM receptor pathway in LUMA, LUMB, HER2, and BASAL BC subtypes. **D** box blots derived from TCGA BRCA dataset showing ID4 expression levels in the different BC subtypes. **E** box blots derived from TCGA BRCA dataset showing expression levels of FOXA1, GATA3, BRCA1, KRT18, KRT8, and KRT19 in the LUMA subtypes expressing high vs. low ID4 expression levels.

High levels of ID4 expression correlate with an aggressive phenotype and a poor prognosis in BLBC, where it directly controls BC cell differentiation, angiogenesis, and BRCA1 activity [18]. However, the role of ID4 in breast cancer cell motility has not yet been explored. Here, we performed GSEA to identify the most correlated pathways to ID4 expression in the TCGA breast cancer dataset using gene sets of the Molecular Signature Database (MSigDB). GSEA analysis showed motility-related pathways, such as Cell adhesion molecules, ECM–receptor interaction, and Focal adhesions among the top-10 ID4-correlated pathways (Fig. 1A, B). Furthermore, hierarchical clustering analysis highlighted that ID4 expression correlates with the above-mentioned motility-related pathways in BLBC subtype, which is characterized by the highest ID4 expression level among all BC subtypes [21] (Fig. 1C, D), and in a subgroup of Luminal-A (LumA) tumors. Besides activation of the motility-related pathways, the subset of LumA tumors presenting high ID4 expression also shows a reduced expression of the luminal commitment factors Foxa1, GATA3, BRCA1, KRT18, KRT8, and KRT19 compared to the portion of LumA expressing low ID4 (Fig. 1E), further confirming the negative association between ID4 and BC cell differentiation. These data suggest that ID4 expression is associated with motility pathways independently of the breast cancer subtype.

### ID4 controls breast cancer cell migratory and invasive ability by inducing focal adhesion formation

**Fig. 2:**
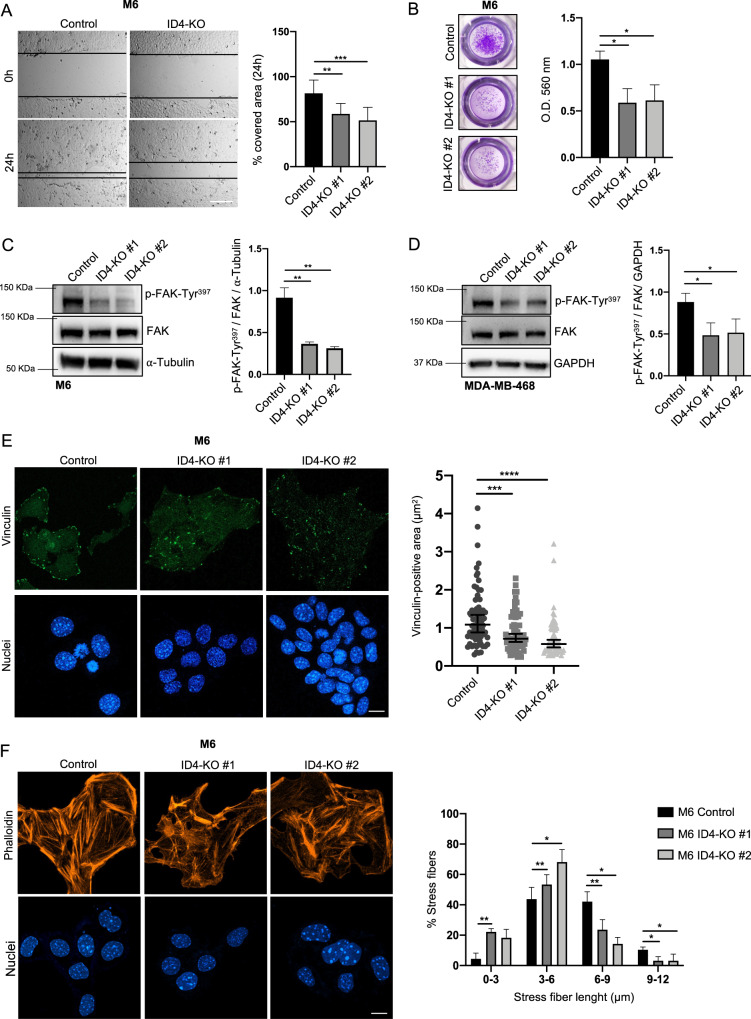
ID4 controls breast cancer cell migratory and invasive ability by inducing focal adhesions formation. **A** wound healing assay performed with M6 Control and ID4-KO cells and graph showing the percentage of covered area at 24 h. Scale bar: 100 µm **B** matrigel invasion assay performed with M6 Control and ID4-KO cells and graph showing the quantification of invaded cells at 24 h. **C** western blot analysis of p-FAK and FAK in M6 Control and ID4-KO cells with the relative quantification graph. **D**: western blot analysis of p-FAK and FAK in MDA-MB-468 Control and ID4-KO cells with the relative quantification graph. **E** immunofluorescence analysis of vinculin in M6 Control and ID4-KO cells with the quantification of vinculin-positive area. **F** immunofluorescence analysis of phalloidin in M6 Control and ID4-KO cells and graph showing the distribution of stress fiber length. Scale bar: 20 µm. Data are presented as mean ± SD. *P < 0.05, **P < 0.01, ***P < 0.001, ****P < 0.0001 calculated by One-way Anova test on *n* = 3 experiments.

To investigate whether ID4 is directly involved in regulating the identified motility-related pathways, we generated ID4 knock-out cells by CRISPR-Cas9-mediated gene editing in the murine M6 and the human MDA-MB-468 BLBC cell lines. We transfected control cells with a guide RNA sequence designed not to target human and mouse genomes. We selected two ID4-KO clones for each cell line based on the absence of ID4 expression analyzed by western blot and RT-qPCR (Supplementary Fig. 1A, B). The ID4-KO cells showed no significant changes in cell morphology, cell proliferation, or cell death compared to control cells (Supplementary Fig. 1C–E). To explore the role of ID4 in cell motility, we first performed a scratch assay using M6 and MDA-MB-468 cells, finding that the percentage of area covered by ID4-KO cells was significantly reduced compared to control cells (Fig. 2A and Supplementary Fig. 2A). Accordingly, the migratory ability analyzed by transwell assay using a chemoattractant was also decreased in M6 ID4-KO cells (Supplementary Fig. 2B). To verify whether lack of ID4 also affects invasive ability, we performed an invasion assay with Matrigel-coated transwells, highlighting a significant reduction in M6 ID4-KO cell invasive ability (Fig. 2B).

Focal adhesions connect the intracellular actin cytoskeleton with the ECM and are critical regulators of cell motility. Considering the positive correlation between ID4 expression and focal adhesion pathway, we next explored whether the migratory defects of ID4-KO cells are linked to alterations in focal adhesion formation and structure. To study FA formation, we analyzed FAK autophosphorylation on Tyr397, characteristic of its activation. The results showed a significant reduction of phospho-FAK expression in both M6 ID4-KO and MDA-MB-468 ID4-KO cells compared to control cells (Fig. 2C, D). To visualize FAs, we performed immunofluorescence staining for vinculin, one of the FA partners recruited by FAK upon its activation. We observed that in both M6 and MDA-MB-468 ID4-KO cells vinculin-positive area was drastically reduced, suggesting an impairment in the formation of the FA (Fig. 2E and Supplementary Fig. 2C). Lack of ID4 in M6 cells also results in shorter stress fiber formation, measured by IF staining with phalloidin, and general disorganization of the F-actin cytoskeleton (Fig. 2F).

### ID4 controls FA formation by stimulating the production of VEGFA in BC cells

**Fig. 3:**
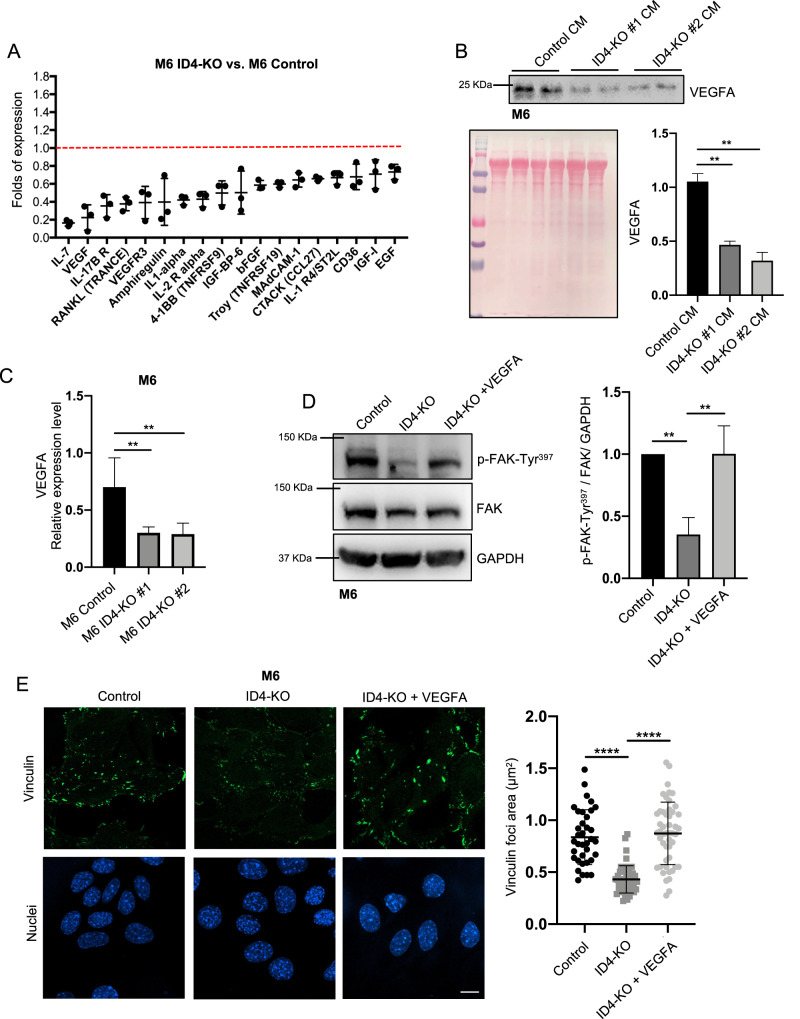
ID4 controls FA formation by stimulating the production and release of VEGFA in BC cells. **A** cytokine array analysis performed with conditioned medium from M6 Control and ID4-KO cells. **B** western blot analysis of VEGFA in conditioned medium from M6 Control and ID4-KO cells with the relative quantification graph. Ponceau S was used for protein normalization. **C** real-time PCR analysis of VEGFA expression in M6 Control and ID4-KO cells. **D** western blot analysis of p-FAK and FAK in M6 Control, ID4-KO, and ID4-KO cells treated with recombinant VEGFA for 24 h, with the quantification graph. **E** immunofluorescence staining of vinculin in M6 Control, ID4-KO, and ID4-KO cells treated with VEGFA for 24 h, and graph showing the quantification of the vinculin-positive area. Scale bar: 20 µm. Data are presented as mean ± SD. **P < 0.01, **** *P* < 0.0001 calculated by One-way Anova test on *n* = 3 experiments.

Several studies reported the strict bidirectional link between focal adhesions and growth factor receptors [24, 25]. Growth factor activation stimulates integrin clustering and the recruitment of FAK, which mediates downstream signaling and stimulates cell migration [26]. Furthermore, integrins can bind directly to growth factors and promote the binding to their receptor and the downstream signaling. We used a cytokine and growth factor array to verify whether the absence of ID4 influences the production and secretion of growth factors and, subsequently, the activation of FA signaling. The array showed that some soluble factors were up-regulated (not shown), and others were down-regulated in the conditioned medium (CM) from M6 ID4-KO cells compared to control cells (Fig. 3A). Among the most downregulated secreted factors, VEGFA captured our attention because several studies reported its ability to control FA activation.

Western blot analysis confirmed the downregulation of VEGFA secretion in the CM from M6 ID4-KO and MDA-MB-468 ID4-KO cells (Fig. 3B and Supplementary Fig. 3A).

We have previously demonstrated that ID4 controls VEGFA isoform splicing in a complex with mutant p53, SRSF1, and Malat1 and that ID4 favors the production of the pro-angiogenic isoform VEGFA165 (VEGFA) over the anti-angiogenic isoform VEGF165b [27]. Therefore, we checked the expression of the pro-angiogenic VEGFA isoform in our CRISPR/Cas9-edited cells M6 ID4-KO and MDA-MB-468 ID4-KO cells by real-time PCR, highlighting a significant reduction of its expression compared to control cells (Fig. 3C and Supplementary Fig. 3B). To verify whether the reduced VEGFA production is responsible for the aberrant FA formation in our system, we treated M6 and MDA-MB-468 ID4-KO cells with recombinant VEGFA at a final concentration of 20 ng/mL and analyzed FAK activation by western blot. The results showed that VEGFA treatment significantly rescued FAK phosphorylation in M6 and MDA-MB-468 ID4-KO cells (Fig. 3D and Supplementary Fig. 3C). Next, to verify whether VEGFA treatment restored FA formation, we cultured M6 ID4-KO cells with recombinant VEGFA and performed an immunofluorescence staining for vinculin. The results showed that VEGFA administration rescues FA area in ID4-KO cells to a level similar to control cells (Fig. 3E). These results indicate that VEGFA production induced by ID4 stimulates FAK phosphorylation and FA formation in BC cells.

### ID4-VEGFA axis promotes BC cell migration by stimulating integrin β3 and VEGFR2 interaction

**Fig. 4:**
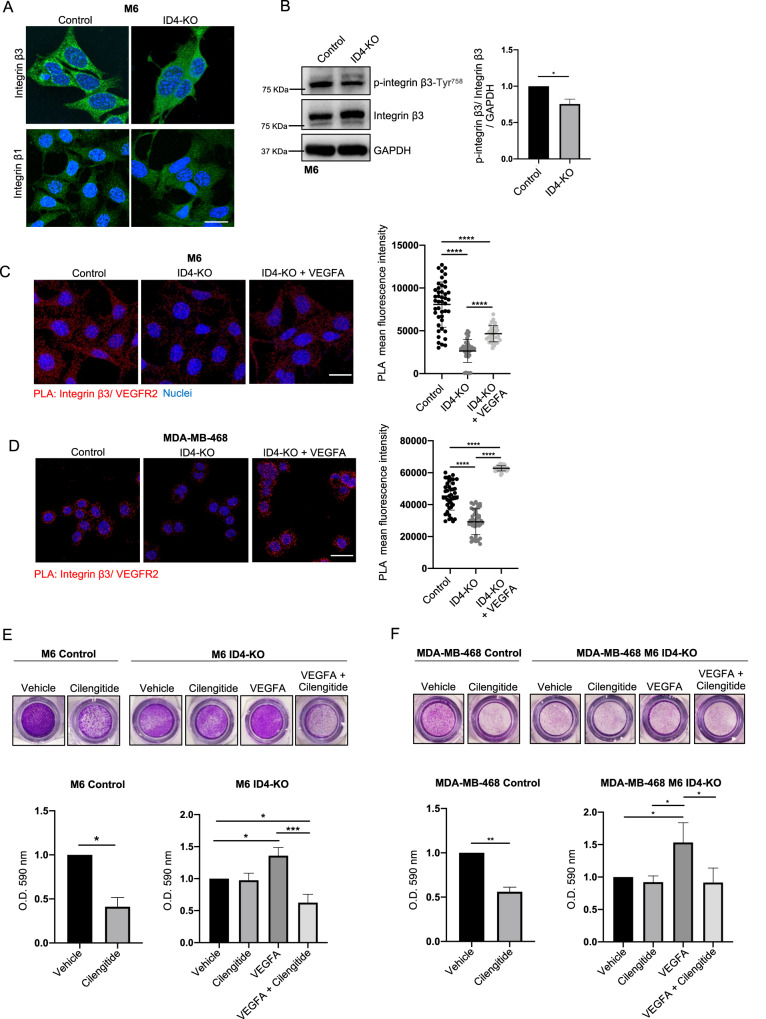
ID4-mediated VEGFA production promotes integrin β3 and VEGFR2 interaction in BC cells. **A** Immunofluorescence staining of integrin β3 and integrin β1 in M6 Control and ID4-KO cells. **B** western blot analysis of p-integrin β3 and integrin β3 in M6 Control and ID4-KO cells, with the relative quantification graph. **C** PLA analysis of integrin β3 and VEGFR2 interaction in M6 Control, ID4-KO, and ID4-KO cells treated with VEGFA for 24 h, with the relative quantification graph. **D** PLA analysis of integrin β3 and VEGFR2 interaction in MDA-MB-468 Control, ID4-KO, and ID4-KO cells treated with VEGFA for 24 h, with the relative quantification graph. **E** transmigration experiment of M6 Control and ID4-KO cells treated with Cilengitide and VEGFA alone or in combination, and quantification of migrated cells at 24 h. **F** transmigration experiment of MDA-MB-468 Control and ID4-KO cells treated with Cilengitide and VEGFA alone or in combination, and quantification of migrated cells at 24 h. Scale bar: 20 µm. Data are presented as mean ± SD. *P < 0.05, **P < 0.01, ***P < 0.001, **** *P* < 0.0001 calculated by Student’s *t* test (**B**, **E**, **F**) or One-way Anova test (**C**, **D**, **E**, **F**) on *n* = 3 experiments.

The best-known function of VEGFA is the promotion of angiogenesis mediated by its binding with VEGFR2, which stimulates endothelial cell migration, survival, and proliferation [28]. In endothelial cells (EC), VEGFR2 interacts with β3 and β1 integrins, such as αvβ3, α9β1, α3β1, and α5β1 to stimulate angiogenesis [29]. For example, VEGFA binding to VEGFR2 triggers VEGFR2 interaction with integrin αvβ3, which is necessary to activate the downstream FAK signaling and stimulate EC migration [30, 31]. Different studies showed that VEGFA acts not only in ECs but also in BC cells to induce an autocrine loop that stimulates cell migration and invasion [32, 33]. BC and EC both express β3 and β1 integrins in tumors [34], thus we wondered whether the reduced secretion of VEGFA in ID4-KO BC cells might influence VEGFR2–integrins interaction and affect the downstream FA formation. We performed immunofluorescence analysis for β3 and β1 integrins in M6 and MDA-MB-468 Control and ID4-KO cells to analyze their localization. We observed that integrin β3 displays a clustered distribution in Control cells, which is lost in ID4-KO cells, suggesting a reduced activation in the absence of ID4. On the contrary, integrin β1 did not show an altered distribution in ID4-KO cells (Fig. 4A and Supplementary Fig. 4A). We then performed a WB analysis for phospho-integrin β3 and found that the phosphorylation levels were significantly reduced in both M6 and MDA-MB-468 ID4-KO cells compared to Control cells, confirming the role of ID4 in integrin β3 activation (Fig. 4B and Supplementary Fig. 4B).

Western blot analysis showed that αv integrin, integrin β3, and VEGFR2 expression levels were not significantly altered in M6 ID4-KO cells compared to Control cells (Supplementary Fig. 4C). However, the colocalization between integrin β3 and VEGFR2 analyzed by confocal IF decreased in the absence of ID4. To verify whether this reduced colocalization was a consequence of reduced VEGFA secretion in ID4-KO cells, we administered recombinant VEGFA at a final concentration of 20 ng/mL to M6 ID4-KO cells and found that integrin β3 and VEGFR2 colocalization was restored (Supplementary Fig. 4D). Next, to confirm the interaction between integrin β3 and VEGFR2, we performed a Proximity Ligation Assay (PLA) in M6 and MDA-MB-468 Control and ID4-KO cells (Fig. 4C, D), and we observed a reduced interaction between the two proteins in the absence of ID4, which increased after culturing the cells with recombinant VEGFA.

To validate the role of VEGFR2 and integrin β3 interaction in FA activation and cell migration, we used Cilengitide, an inhibitor of integrin αvβ3 activation [35], with or without concomitant VEGFA treatment. We treated M6 and MDA-MB-468 cells with Cilengitide at 0.1 μM for 24 h, a concentration that did not alter cell number, and performed a transwell migration experiment using FBS as a chemoattractant. We observed that Cilengitide treatment reduced migration only in Control cells and not in ID4-KO cells (Fig. 4E, F). Moreover, we observed that pretreatment of M6 or MDA-MB-468 ID4-KO cells with recombinant VEGFA for 30 min prior to the experiment, increased cell migration compared to untreated cells. Pretreatment was necessary because the presence of VEGFA during the experiment contrasted with the chemoattractant activity of FBS. Finally, to investigate the role of VEGFR2 and integrin β3 interaction in cell migration, we pre-treated M6 or MDA-MB-468 ID4-KO cells with VEGFA to reactivate VEGFR2 and integrin β3 interaction and then performed transwell migration assay in the presence of Cilengitide. In this case, we observed a reduction in cell migration compared to ID4-KO cells treated with VEGFA alone (Fig. 4E, F). Moreover, Cilengitide treatment reduced FAK phosphorylation in M6 Control cells but not in M6 ID4-KO cells (Supplementary Fig. 5A). These results suggest that ID4 controls cell migration by VEGFA-mediated integrin β3–VEGFR2 interaction.

We also validated these results in a primary murine cell line where we stably overexpressed ID4 (Supplementary Fig. 5B). In line with the previous results, ID4 overexpression was associated with an increased FAK phosphorylation, and an increased cell migratory ability compared to Control cells, while Cilengitide treatment reduced migration and FAK phosphorylation of ID4 overexpressing cells (Supplementary Fig. 5C, D).

Finally, to investigate the effect of ID4/VEGFA axis activation on pathways other than FA, we analyzed the activation of AKT, p38, and ERK 1/2, which are typically downstream of VEGFA signaling [28]. We performed WB analysis of phospho-AKT, phospho-p38, and phospho-ERK in M6 Control and ID4-KO cells treated or not with recombinant VEGFA for 24 h. We observed a significant reduction of AKT phosphorylation in the absence of ID4, which was rescued after VEGFA addition. On the contrary, phospho-p38 increased in ID4-KO cells and decreased after VEGFA addition. As reported in literature, several studies showed that AKT inhibits p38 activation and, in parallel, p38 negatively affects AKT activity [36], justifying the opposite trend we observed. Finally, we did not observe differences in ERK 1/2 phosphorylation between Control and ID4-KO cells. However, we did observe an increased ERK 1/2 phosphorylation in ID4-KO cells after the addition of recombinant VEGFA, which might be due to the activation of other VEGFA-dependent pathways. In conclusion, ID4/ VEGFA/ VEGFR2 axis affects AKT and p38 pathway activation in M6 cells (Supplementary Fig. 6).

### ID4-VEGFA axis controls YAP/TAZ nuclear localization and pathway activation in BC cells

**Fig. 5:**
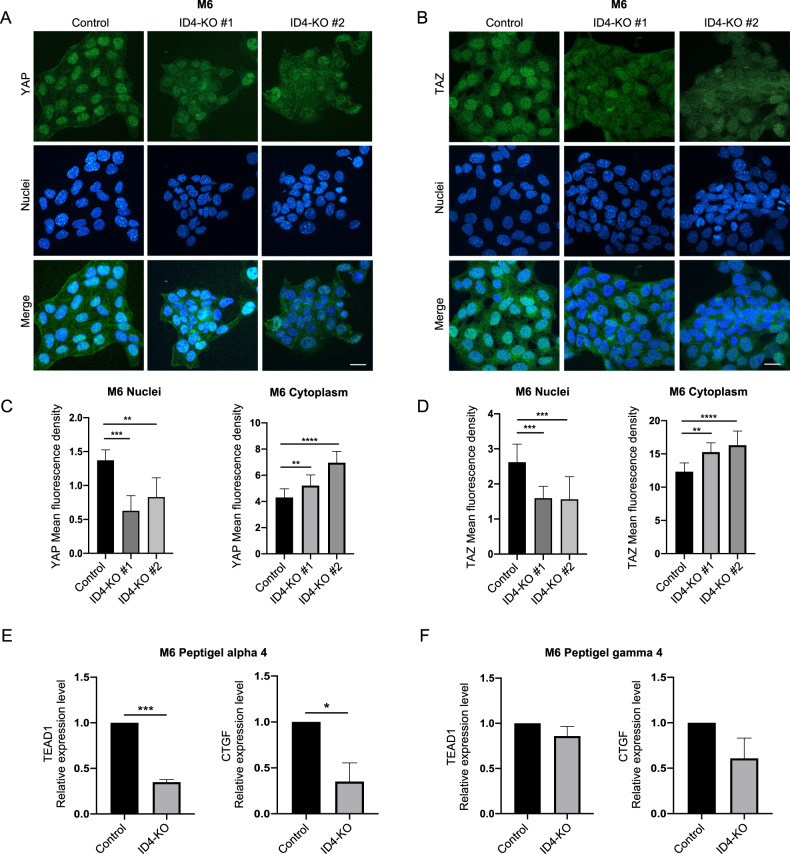
ID4-KO BC cells display reduced YAP/TAZ nuclear localization and activation. **A** immunofluorescence analysis of YAP in M6 Control and ID4-KO cells. **B** immunofluorescence analysis of TAZ in M6 Control and ID4-KO cells. **C** quantification of YAP mean fluorescence intensity in the nuclei and cytoplasm of M6 Control and ID4-KO cells. **D** quantification of TAZ mean fluorescence intensity in the nuclei and cytoplasm of M6 Control and ID4-KO cells. **E** real-time analysis of TEAD1 and CTGF in M6 Control and ID4-KO cells grown in peptigel alpha 4. **F** real-time analysis of TEAD1 and CTGF in M6 Control and ID4-KO cells grown in peptigel gamma 4. Scale bar: 20 µm. Data are presented as mean ± SD. *P < 0.05, **P < 0.01, ***P < 0.001, ****P < 0.0001 calculated by One-way Anova (**C**, **D**) or Student’s *t* test (**E,** **F**) on *n* = 3 experiments.

The activation of the integrin-focal adhesion signaling stimulates YAP and TAZ activation. YAP and TAZ are inactive when localized in the cytoplasm, but after nuclear translocation, they associate with TEAD transcription factors and induce the expression of downstream targets [14]. Therefore, we wondered whether the activation of the FA pathway induced by ID4-VEGFA axis could influence YAP/TAZ activity. To investigate this possibility, we performed immunofluorescence analysis for YAP and TAZ in M6 and MDA-MB-468 ID4-KO cells to analyze their localization. The results showed a significant decrease in the nuclear mean fluorescence intensity in ID4-KO cells and an increase in the cytoplasmic signal (Fig. 5A–D and Supplementary Fig. 7B). This result suggests that ID4 might control YAP/TAZ nuclear shuttling. To exclude that ID4 influences YAP/TAZ expression levels, we analyzed YAP and TAZ mRNA and protein levels in M6 and MDA-MB-468 cells, highlighting no significant differences compared with the control cells (Supplementary Fig. 8). Next, we verified whether the reduced nuclear YAP and TAZ translocation influences the transcription levels of some target genes. Therefore, we analyzed two important YAP/TAZ-related genes: CCN2 (CTGF) and the YAP binding partner TEAD1. High ECM stiffness is fundamental for activating YAP/TAZ signaling cascade, so we decided to grow M6 Control and ID4-KO cells in hydrogel peptigel matrixes, which simulate different levels of ECM stiffness. We found that growing M6 ID4-KO cells in a stiffer matrix (peptigel alpha4) led to a reduction of CTGF and TEAD1 expression analyzed by real-time PCR, compared to control cells (Fig. 5E). Contrarily, growing M6 ID4-KO cells in a softer matrix (peptigel gamma4), CCN2, and TEAD1 expression levels did not decrease significantly (Fig. 5F). YAP/TAZ expression and activation are also positively correlated to the cell adhesion area and cell volume [37, 38]. Therefore, we analyzed the total area of occupancy of M6 Control and ID4-KO cells by label-free Dynamic mass redistribution, observing that it was reduced in ID4-KO cells (Supplementary Fig. 7A).

**Fig. 6:**
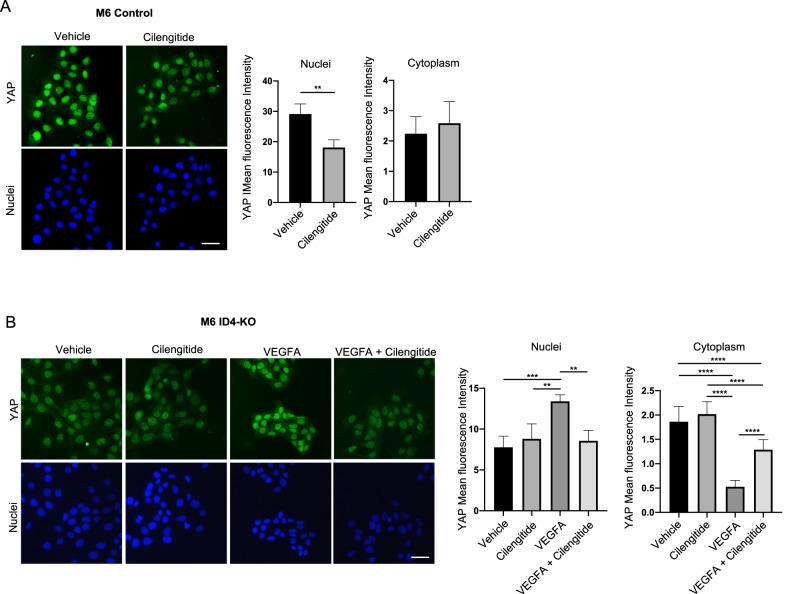
ID4/VEGFA axis stimulates nuclear YAP localization via integrin β3/VEGFR2 signaling in BC cells. **A** immunofluorescence analysis of YAP in M6 Control cells treated with vehicle or Cilengitide at 0.1 µM, with the relative mean fluorescence intensity quantification in nuclei and cytoplasm. **B** immunofluorescence analysis of M6 ID4-KO cells treated with vehicle, Cilengitide, and/or VEGFA, with the relative mean fluorescence intensity quantification in nuclei and cytoplasm. Scale bar: 50 µm. Data are presented as mean ± SD. **P < 0.01, ****P < 0.0001 calculated by Student’s *t* test (**A**) or One-way Anova (**B**) on *n* = 3 experiments.

Next, we investigated whether YAP/TAZ nuclear localization was stimulated by VEGFA/VEGFR2/integrin β3 pathway. To explore this possibility, we treated M6 or MDA-MB-468 Control and ID4-KO cells with Cilengitide and/or recombinant VEGFA at the same doses indicated above and analyzed YAP localization by immunofluorescence. We observed that inhibition of integrin β3 activity with Cilengitide treatment decreased YAP nuclear fluorescence in Control cells but not in cells lacking ID4 (Fig. 6A, B, and Supplementary Fig. 7C, D). Treatment of M6 or MDA-MB-468 ID4-KO cells with recombinant VEGFA instead, rescued YAP nuclear fluorescence, and double treatment with Cilengitide and recombinant VEGFA decreased the nuclear fluorescence of ID4-KO cells to a level similar to that of untreated cells (Fig. 6B and Supplementary Fig. 7D). These results together suggest that ID4-mediated VEGFA production stimulates YAP nuclear localization via VEGFR2/integrin β3 signaling.

### ID4 correlates with nuclear YAP/TAZ and with YAP/TAZ signatures in BC patients

**Fig. 7:**
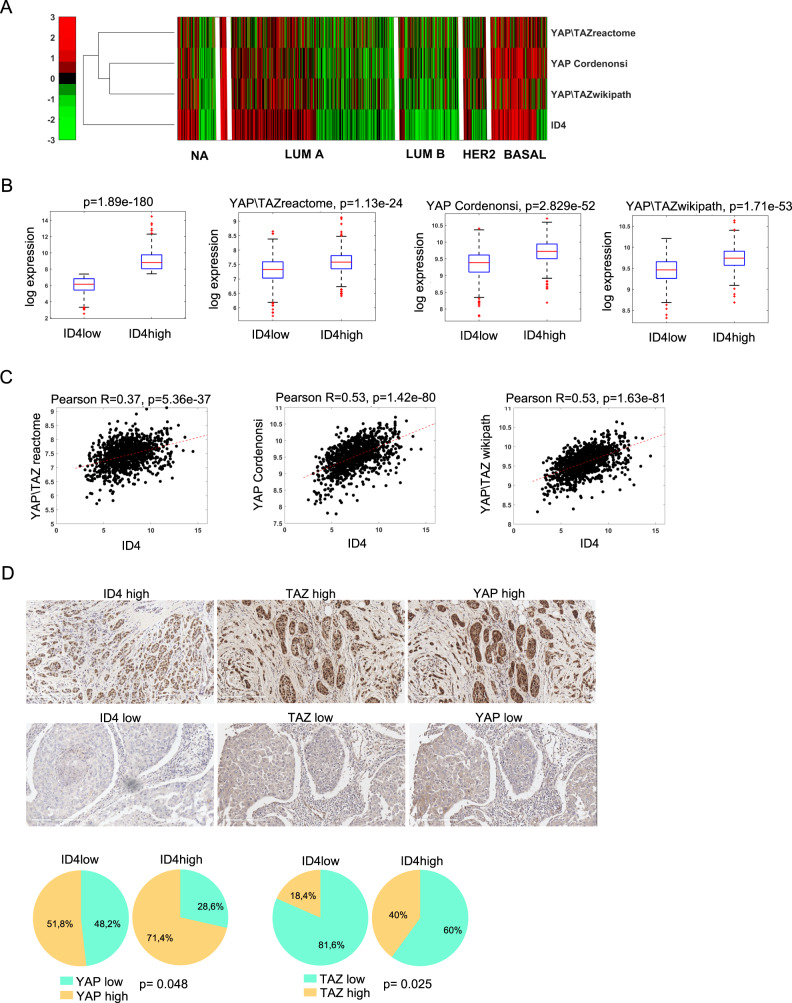
ID4 correlates with nuclear YAP/TAZ and with YAP/TAZ signatures in BC patients. **A** heatmap derived from TCGA BRCA dataset showing ID4 expression and the activation of three YAP/TAZ signatures from MsigDB in LUMA, LUMB, HER2 and BASAL breast cancer subtypes. **B** box plots from TCGA BRCA dataset showing the expression levels of three YAP/TAZ signatures in ID4 high vs ID4 low breast cancers. **C** graphs derived from TCGA BRCA dataset showing the correlation between three different YAP/TAZ signatures and ID4 expression in breast cancer. **D** immunohistochemistry staining of ID4, YAP, and TAZ in tissue microarrays of TNBC, and pie charts showing the percentages of cells expressing high or low nuclear YAP/TAZ staining in tissues expressing high or low ID4. Statistical analysis of TMA data was performed by Fisher’s exact test.

We next explored whether ID4 expression correlates with YAP/TAZ signaling in human breast cancer patients. Therefore, we analyzed ID4 expression and the activation of three YAP/TAZ signatures of the TCGA cohort in different subtypes of breast cancer. As shown in Fig. 7A, B, and in the correlation graphs in Fig. 7C, ID4 expression levels strongly correlate with all the three YAP/TAZ signatures analyzed, independently of the breast cancer subtype. To further characterize this association, we stained by IHC TNBC tissue microarrays for YAP/TAZ and ID4 and analyzed their correlation. We classified the expression level of YAP, TAZ, and ID4 with a score from 0 to 3 and divided the samples into “high” or “low” depending on the score. Interestingly, we found that high YAP and TAZ nuclear staining significantly correlated with high ID4 staining, confirming the association between ID4 and YAP/TAZ activation (Fig. 7D). These results together indicate that ID4 expression correlates with the nuclear localization of YAP/TAZ and with the activation of YAP/TAZ -related pathways in primary breast cancers.

## Discussion

Crosstalk between the major angiogenic growth factor VEGFA and integrin cell adhesion receptors has recently emerged as a critical factor in regulating angiogenesis and tumor development [29, 39]. Beyond angiogenesis, VEGFA also controls the behavior of BC cells and other cells in the tumor microenvironment [32, 40]. For example, in MDA-MB-231 BC cells, which do not express VEGFR2, VEGFA binds NRP1 receptor controlling cell morphology and migration through Cdc42-mediated filopodia formation [41]. Moreover, in MDA-MB-231 and SUM149PT BC cell lines, VEGFA induces metastasis formation and stemness by upregulating Sox2 [42].

Here, we showed that ID4 protein expression in BC contributes to tumor aggressiveness stimulating cell migration and invasion by regulating VEGFA production. Correlation analysis performed in BC patients showed that ID4 expression strongly correlates with motility pathways, such as Focal Adhesions, Cell Adhesion Molecules, and ECM receptor interaction. The dysregulation of these pathways has also been linked to collective cancer cell migration during metastasis formation and adhesion-independent growth [43, 44]. The in-vitro experiments we performed confirmed that FA formation and cell migration/ invasion were compromised in BC cells lacking ID4.

ID4 expression and prognostic power are associated almost exclusively with basal-like breast cancer. However, our analysis highlighted that the association between ID4 expression and the motility-related pathways is also present in a subset of LumA with high ID4 and lower expression of differentiation markers. A recent study highlighted the close association between the basal and the LumA subtypes, demonstrating that the inhibition of LATS1/2 in luminal BC cells leads to the acquisition of a basal-like phenotype [45]. Therefore, ID4 expression might discriminate LumA breast cancers with more aggressive phenotypes.

Several studies showed that focal adhesion activation is regulated, among other factors, by growth factors binding to their receptors, such as EGF/EGFR and VEGF/VEGFR2, on endothelial cells [46–48]. In endothelial cells, VEGFA binding to VEGFR2 stimulates its interaction with integrin αvβ3 driving FAK activation and angiogenesis [30, 31, 49]. Mechanistically, in ECs VEGFA stimulates c-Src-mediated integrin β3 phosphorylation, increasing the affinity of β3 integrin and VEGFR2 binding. Replacing functional integrin β3 with an inactive one blocked the interaction with VEGFR2 and its activation, compromising integrin β3 capacity to respond to VEGFA [39, 50]. Here, we demonstrated that the secretion of VEGFA induced by ID4 stimulates the interaction between VEGFR2 and integrin β3 in BC cells, promoting FAK phosphorylation, the formation of focal adhesions, and inducing cell migration and invasion. Interestingly, we found that the ID4/VEGFA axis in BC cells stimulates AKT phosphorylation while reducing p38 phosphorylation, which might contribute to the observed phenotype. These results suggest the possibility of using VEGFR2 or integrin β3 inhibitors to interfere with BC metastasis formation.

The development of new VEGFR2 inhibitors has recently gained interest for treating TNBC, as these inhibitors displayed potent cytotoxic and antiproliferative effects in vitro [51, 52]. Furthermore, direct inhibition of VEGFR2 might overcome receptor activation mediated by ligands different from VEGF, such as HIV-1-Tat and gremlin [53].

The activation of integrin β3 is fundamental for MDA-MB-231 cell bone metastasis formation, and its inhibition downregulates genes involved in multiple tumor pathways, such as angiogenesis, migration, proliferation, and metabolism [54]. Integrin β3 expression also increases in breast cancer after chemotherapy, underlining its importance in tumor progression [55]. However, although preclinical testing of integrin inhibitors has shown promising results, clinical trials led to poor outcomes due to the development of resistance mechanisms and the complexity of integrin pathways [51, 56]. The development of novel inhibitors and their combination with other therapies might be the key to target integrin pathways successfully.

Finally, we showed that the ID4-dependent activation of the VEGFA/ VEGFR2/integrin β3 pathway induces YAP/TAZ nuclear translocation and activation in BC cells. The staining we performed on TNBC TMA showed a strong correlation between ID4 expression and YAP/TAZ pathway activation. This correlation was present in the basal-like subtype and again in the LUMA subtype fraction expressing high ID4 levels. Considering that YAP/TAZ pathway activation confers basal-like characteristics to Luminal cells, ID4-mediated YAP/TAZ activation may contribute to the basal-like and LUMA phenotype plasticity.

In conclusion, our work supports the evidence that interfering with VEGFA/ VEGFR2/ integrin β3 pathway can directly affect breast cancer cell migration beyond the largely explored anti-angiogenic function. Moreover, targeting this pathway can counteract tumorigenesis by indirectly interfering with YAP/TAZ activation.

## Materials and methods

### Cell culture and generation of kcnock-out cells

M6 (ATCC CRL-3441) and MDA-MB-468 (ATCC HTB-132) cell lines were grown in DMEM containing 10% heat-inactivated FBS serum, 1% penicillin/streptomycin and 1% L-glutamine, at 37 °C and 5% CO_2_.

M6 and MDA-MB-468 ID4-Knock Out cells were generated by CRISPR-Cas9 gene editing. Alt-R® S.p. HiFi Cas9 Nuclease V3 (Cat No. 1081060) and Alt-R® CRISPR-Cas9 tracrRNA, ATTO™ 550 (Cat. No. 1075928) were purchased from IDT (Coralville, Iowa, USA). The following predesigned guide RNA were used for human cells: Hs.Cas9.ID4.1.AA and Hs.Cas9.ID4.1.AD. For mouse cells, the following predesigned guide RNA was used: Mm.Cas9.ID4.1.AA, Mm.Cas9.ID4.1.AB. Transfection of RNP complex was performed according to the manufacturers’ protocols at a final concentration of 10 nM.

After 2 days, the cells were detached and plated into a 96-well plate at a density of 1 cell/well. Single clones were expanded and checked for ID4 expression by real-time PCR and western blot analysis.

Treatments with recombinant VEGFA were performed at a final concentration of 20 ng/mL.

Sal21 cells were cultured in DMEM-F12 (Sigma) supplemented with 10% FBS, 1% P/S, and insulin (Sigma I9278). For ID4 overexpression, Sal21 cells were transfected with mouse ID4 overexpressing plasmid (MR227565, Origene, Rockville, MD, USA) and the respective control vector, using Lipofectamine 2000 (Thermo Fisher) according to the manufacturer’s protocol. After 48 h, G418 (A1720 Sigma-Aldrich) was added to the culture medium at a concentration of 1000 µg/mL to select transfected cells. Cells were maintained under G418 selection for 3 weeks before performing experiments.

### Bioinformatic analysis

Standardized TCGA data were obtained from the Broad Institute TCGA Genome Data Analysis Center (2016, 10.7908/C11G0KM9). Differential expression of genes among subgroups of samples was evaluated by two-sided Wilcoxon rank sum test or KruskalWallis test. *P* values less than 0.05 were considered significant. A Gene Set Enrichment Analysis (GSEA software; https://www.gsea-msigdb.org/gsea/index.jsp) was conducted by using the curated gene sets of the MSigDB derivated from KEGG and Hallmark collections. GSEA was run in preranked mode using classic as metric and 1000 permutations. Hierarchical Clustering was built by using Euclidean distance and average linkage among mean expression of genes included in the core enrichment of each pathway. A Pearson’s correlation coefficient was evaluated between ID4 expression and mean expression of specific pathways. All the analyses were conducted with MATLAB R2022a.

### Statistical analysis

All data showed in graphs were expressed as means ± standard deviation (SD). All the experiments were performed in triplicate. Differences between three or more groups were assessed by One-way ANOVA test with adjustment for multiple comparisons. Differences between two groups were assessed by two-tailed Student’s unpaired *t* test. A *p* value < 0.05 was considered statistically significant. Statistical analyses were performed using Prism 8 (GraphPad, San Diego, USA).

### Migration and invasion assays

For the wound-healing assay, a confluent monolayer of cells was scratched with a 10 μl pipette tip and rinsed twice with PBS. The cells were maintained in a serum-free medium and analyzed for migration 24 h or 72 h later. The percentage of wound closure was quantified using the open-source imageJ/Fiji software (Bethesda, Maryland, USA).

For the transwell migration assay, 50,000 cells were plated in an 8-micron pore size transwell in serum-free medium and placed into a 24-well plate with 10% FBS for 24 h. The interior of the transwell was cleaned with a cotton swab and the transwells were immersed in crystal violet for 10 min RT, then washed in water and air dried. For quantification, stained cells were either counted or eluted in 33% acetic acid solution for 10 min RT. The eluent absorbance was read at 590 nm with a plate reader.

For invasion assay 8-micron pore size transwells were coated with 0.1 mL Matrigel (Corning, Corning, NY, USA) diluted in coating buffer (0.01 M Tris-HCl pH 8, 0.7% NaCl) at a final concentration of 250 μg/μl. Then, 200,000 cells were plated on Matrigel in serum-free medium and the transwells were placed into a 24-well plate with 10% FBS for 24 h. Quantification of migrated cells was performed as described for migration assay.

### Western blot

For western blot analysis, cells were lysed in RIPA buffer (50 mM Tris-HCl pH 8.0, 150 mM NaCl, 0.1% Triton X-100, 0.5% sodium deoxycholate 0.1% SDS, 1 mM sodium orthovanadate, 1 mM NaF, Protease inhibitors) and then mixed in 4× Laemmli buffer. Protein concentration was measured using a Bio-Rad protein assay kit (Bio-Rad Laboratories, Hercules, CA, USA). Total protein extracts were run on polyacrylamide gel and then transferred onto nitrocellulose membrane. For the analysis of secreted VEGFA 90% confluent cells were grown for 24 h or 48 h in serum-free medium. Then, proteins contained in the medium were precipitated with Tricholoacetic Acid (TCA). Briefly, 100 μl of 100% TCA were added to 1 mL of medium, and proteins were precipitated on ice for 30 min. Next, samples were centrifuged at 10,000 × *g* for 15 min at 4 °C and the pellet was washed three times with ice-cold acetone by centrifugation at 10,000 × *g* for 5 min. Finally, the pellet was allowed to air-dry and resuspended in 2× Laemmli buffer for WB analysis. Ponceau S was used to quantify total proteins from the conditioned medium.

The following primary antibodies were used: ID4 (B5) (sc-365656), GAPDH (sc-32,233), VEGFR2 (sc-6251), p-Integrin β3 (sc-136458) (Santa Cruz Biotechnology, Dallas, TX, USA); p-FAK (Tyr397) (3283), FAK (3285), integrin β3 (13166), integrin αV (4711), YAP (14074), TAZ (83669), p-AKT (9271), AKT (9272), p-p38 (9211), p38 (9212), p-ERK 1/2 (9102), ERK 1/2 (9102) (Cell Signaling Technology, Danvers, MA, USA); VEGFA (ab46154) (Abcam, Cambridge, UK), alpha-Tubulin (Sigma, St. Louis, MO, USA). A secondary antibody fused with horseradish peroxidase was used for chemiluminescence detection on a UVITEC instrument (Uvitec, Cambridge, UK).

### RNA isolation and real-time PCR

Total RNA was isolated with TRIzol (Sigma), and its concentration was measured using a NanoDrop 2000 (NanoDrop Technologies, Wilmington, DE, USA). Reverse transcription was performed with MMLV-RT (Invitrogen, Whaltam, MA, USA). Real-time PCR was performed on ABI PRISM 7500 Fast Sequence Detection System (Applied Biosys- tems, Carlsbad, CA, USA). The expression values of mRNAs were calculated by standard curve method and normalized over H3 housekeeping control gene. Primers are listed in Supplementary Table 1. *P* values were calculated with two-tailed Student’s *t* test. Statistically significant results were referred with a *P* value < 0.05.

### Immunofluorescence

For immunofluorescence, cells were cultured on a cover glass and fixed in 4% formaldehyde in PBS, then permeabilized for 10 min with 0.1% Triton X-100 in PBS and blocked for 30 min with BSA 4% in PBS. Incubation with primary antibodies for VEGFR2, integrin β3, YAP, TAZ, Vinculin (sc-73614 Santa Cruz Biotechnology) was performed overnight at 4 °C in a humidified chamber in BSA 1% in PBS. Incubation with secondary antibodies was performed for 1 h at RT in a humidified chamber in BSA 1% in PBS with Goat anti-rabbit Alexa Fluor 488 (A-11008 Thermo Fisher), Goat anti-mouse Alexa Fluor 488 (A-11001 Thermo Fisher) Goat anti-mouse Alexa Fluor 555 (A-21422 Thermo Fisher). Rhodamine Phalloidin (R415 Thermo Fisher) staining was performed together with secondary antibodies. Nuclei were stained with Hoechst and the slides were mounted with glycerol mounting medium. Images were acquired under a Zeiss LSM 900 confocal microscope (Zeiss, Oberkochen, Germany), and images were analyzed with ImageJ software.

### Immunohistochemistry

The immunohistochemical assessment of 127 cases of triple-negative BC on TMA (TMA #BR1301 from US Biomax, Rockville, MD, USA) was performed with the following antibodies: ID4, YAP (H-9): sc-271134 (Santa Cruz), TAZ (M2-616) 560235 (BD Pharmigen, Franklin Lakes, NJ, USA) overnight incubation at the dilution of 1:100. Immunoreactions were revealed by Bond Polymer Refine Detection System on an automated auto-stainer (Bond IIITM Stainer, Leica Biosystem, Milan, Italy). Whole slide images of IHC were acquired by scanning of the original glass slides using Aperio CS2 whole slide scanner (Leica Biosystems, Germany). The default autofocus mode was used, but in a few cases, manual focus was applied. The image files (.svs format) were stored on a Network Attached Storage running with the Aperio ScanScope software. Staining intensity for ID4 was evaluated as: 0 negative, 1+ weak, 2+ moderate, 3+ strong. ID4 was considered negative when less than ≤5% of the neoplastic cells exhibited nuclear immunoreaction. Staining intensity for YAP and TAZ was evaluated as: 0 negative, 1+ weak, 2+ moderate, 3+ strong as a percentage of positive neoplastic cells. Percentage of cells expressing YAP/TAZ in nucleus or cytoplasm was also calculated. Differences in the percentages of ID4 and YAP/TAZ in the nuclear region were evaluated by Fisher’s exact test setting a threshold to the 75° percentile of the ID4 distribution.

### 3D cell culture in PeptiGels

To grow cells in peptigel hydrogels (Manchester Biogel, Mereside, UK) manufacturer’s protocol was followed. Briefly, cells diluted in complete culture medium were mixed with peptigel at 1:5 ratio. Then, 100 μL of the mixture were dispensed into an insert placed in a 24-well plate, and cells were grown for 6 days to allow the formation of 3D structures.

### Cytokine array

Cytokine array was performed with Mouse Cytokine Array G2000 Kit (RayBiotech, Peachtree Corners, GA, USA). Briefly, 100 μL of conditioned medium from confluent cells grown for 24 h with serum-free medium were incubated with cytokine array membranes. Incubation was performed according to the manufacturer’s protocol. Array scanner and analysis were performed by RayBiotech Service, and data were normalized on control cells.

### Proximity ligation assay

The Duolink PLA was used (Sigma-Aldrich) to study VEGFR2 and integrin β3 interaction. Cells were cultured on a cover glass and fixed in 4% formaldehyde in PBS, then permeabilized for 10 min with 0.1% Triton X-100 in PBS and blocked for 30 min with Duolink blocking buffer. Further, the manufacturer’s protocol was followed. Nuclei were stained with Hoechst and the slides were mounted with glycerol mounting medium. ImageJ software was used to quantify mean fluorescence intensity on a number of 40 cells per condition.

### Supplementary information

Supplementary Materials and Methods

Supplementary Figure 1

Supplementary Figure 2

Supplementary Figure 3

Supplementary Figure 4

Supplementary Figure 5

Supplementary Figure 6

Supplementary Figure 7

Supplementary Figure 8

aj-checklist

## Data Availability

The published article includes all data generated/analyzed for this study. Other relevant data are available from the authors upon request.
