# Supplementary material for: ID4-dependent secretion of VEGFA enhances the invasion capability of breast cancer cells and activates YAP/TAZ via integrin β3-VEGFR2 interaction
Source: Cell Death Dis. 2024 Feb 6;15(2):113. doi: 10.1038/s41419-024-06491-2 (PMC10847507; doi:10.1038/s41419-024-06491-2)
Supplement: Supplementary file 1 — Supplementary Materials and Methods [file 41419_2024_6491_MOESM1_ESM.docx]

**Supplementary Materials and Methods**

**Proliferation assay**

Proliferation assay was performed by flow cytometry analysis with eFluor™ 670 dye (Thermo Fisher). Briefly, cells were detached and stained in 5 μM eFluor in PBS for 10 min at 37 °C in the dark. Labeling was stopped by adding 4 times complete culture medium and incubating on ice for 5 min. Cells were cultured for 48h in complete medium. For the analysis, cells were detached with trypsin, pelleted, and resuspended in PBS 2% FBS. Flow cytometry analysis was performed with CytoFlex (Beckman Coulter, Brea, CA, USA).

**Cell death assay**

Cell death was analyzed by flow cytometry with SYTOX™ Blue Dead Cell Stain (Thermo Fisher). Briefly, cells were harvested with trypsin, stained with 1 μM SYTOX Blue in PBS 2% FBS for 5 min RT in the dark and immediately analyzed. Flow cytometry was performed with CytoFlex (Beckman Coulter).

**Wright-Giemsa staining**

Cells were grown on a coverslip, stained with Wright-Giemsa (Sigma-Aldrich) for 3 min, and then washed with deionized water for 10 min and air dried before evaluation.

**Dynamic mass redistribution (DMR) label-free assay**

To evaluate the space occupied by cultured cells, a Dynamic mass redistribution (DMR) label-free assay was performed by culturing cells for 72h. The analysis was performed at different timepoints by scanning the plates with an EnSpire Multimode Plate Reader (PerkinElmer, Waltham, MA, USA).

| Primers List |  |  |  |  |
| --- | --- | --- | --- | --- |
| ID4 (mouse and human) |  | For: GTGCGATATGAACGACTGCT  Rev: CAGGATCTCCACTTTGCTGA |  |  |
| VEGF165 TaqMan (human) |  | For: CGCAGACGTGTA AATGTTCCT  Rev: GCC TCG GCT TGT CAC ATC  FAM probe: CAAGGCGAGGCAGCTTGAGTTAAA |  |  |
| VEGF164 TaqMan (mouse) |  | For: CGCAGACGTGTA AATGTTCCT  Rev: TCACCGCCTCGGCTTGTCACAT  FAM probe: CAAGGCGAGGCAGCTTGAGTTAAA |  |  |
| H3 (mouse and human) |  | For: GTGAAGAAACCTCATCGTTACAGGCCTGGT  Rev: CTGCAAAGCACCAATAGCTGCACTCTGGAA |  |  |
| YAP (mouse) |  | For: TACTGATGCAGGTACTGCGG  Rev: TCAGGGATCTCAAAGGAGGAC |  |  |
| YAP (human) |  | For: GCAAATTCTCCAAAATGTCAGG  Rev: CGGGAGAAGACACTGGATTT |  |  |
| TAZ (mouse and human) |  | For: GAAGGTGATGAATCAGCCTCT  Rev: GTTCTGAGTCGGGTGGTTCTG |  |  |
| CTGF |  | For: CCACCCGAGTTACCAATGAC  Rev: GTGCAGCCAGAAAGCTCA |  |  |
| TEAD1 |  | For: TCCGCTTTCCTTGAACAGCAGAG  Rev: GGGTCACTGTAAGAATGGTTGGC |  |  |
