# Supplementary material for: ID4-dependent secretion of VEGFA enhances the invasion capability of breast cancer cells and activates YAP/TAZ via integrin β3-VEGFR2 interaction
Source: Cell Death Dis. 2024 Feb 6;15(2):113. doi: 10.1038/s41419-024-06491-2 (PMC10847507; doi:10.1038/s41419-024-06491-2)
Supplement: Supplementary file 2 — Supplementary Figure 1 [file 41419_2024_6491_MOESM2_ESM.pdf]

Supplementary figure 1

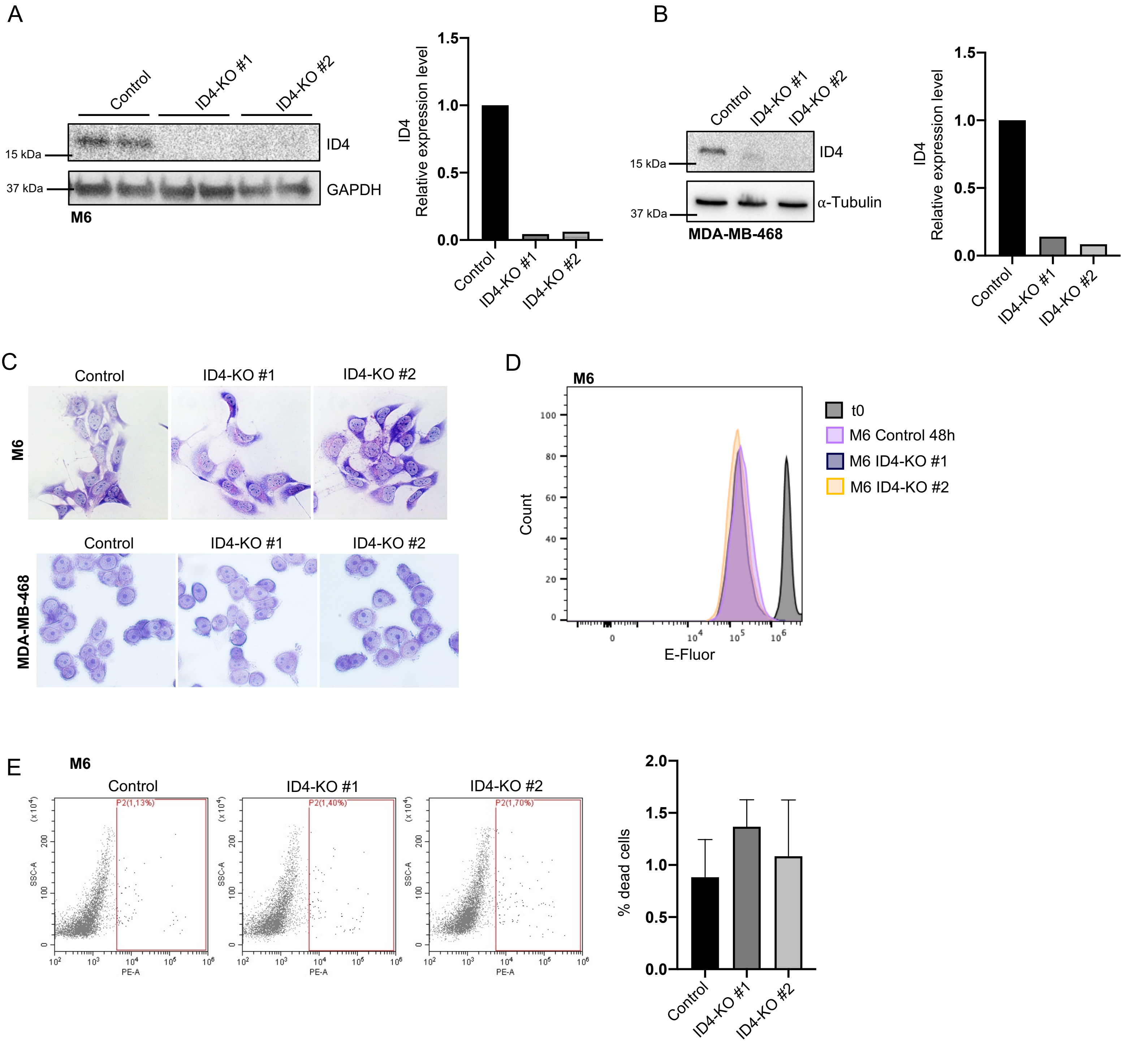

Supplementary figure 1. A: western blot analysis of ID4 in M6 Control and ID4-KO cells, and quantification graph. B: western blot analysis of ID4 in MDA-MB-468 Control and ID4-KO cells, and quantification graph. C: Giemsa staining of M6 and MDA-MB-468 Control and ID4-KO cells. D: flow cytometry analysis to evaluate proliferation by e-Fluor staining in M6 Control and ID4-KO cells, at 48h of culture. E: flow cytometry analysis with Sytox blue to evaluate cell death in M6 Control and ID4-KO cells, n= 3 experiments.
