# Supplementary material for: ID4-dependent secretion of VEGFA enhances the invasion capability of breast cancer cells and activates YAP/TAZ via integrin β3-VEGFR2 interaction
Source: Cell Death Dis. 2024 Feb 6;15(2):113. doi: 10.1038/s41419-024-06491-2 (PMC10847507; doi:10.1038/s41419-024-06491-2)
Supplement: Supplementary file 3 — Supplementary Figure 2 [file 41419_2024_6491_MOESM3_ESM.pdf]

Supplementary figure 2

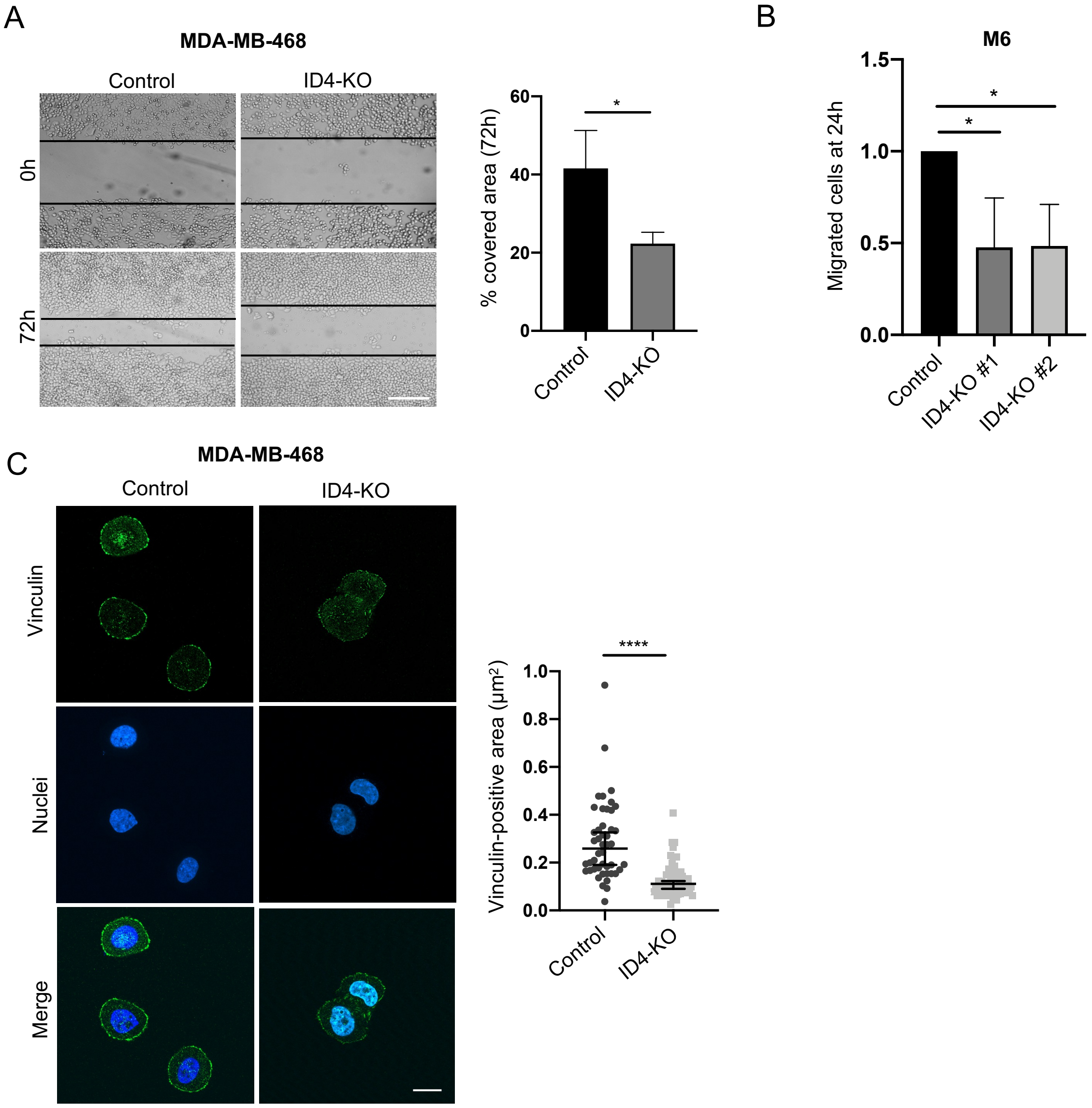

Supplementary Figure 2. A: wound healing assay performed with MDA-MB-468 Control and ID4-KO cells and graph showing the percentage of covered area at 72h. Scale bar: 100 µm. B: quantification of transwell migration experiment of M6 Control and ID4-KO cells at 24h. C: evaluation of vinculin-positive area in MDA-MB-468 Control and ID4-KO by immunofluorescence staining. Scale bar: 20 µm. Data are presented as mean ± SD. *P < 0.05, **P < 0.01, ***P < 0.001, **** P < 0.0001 calculated by One-way Anova (B) or Student's t-test (A and C) on n=3 experiments.
