# Supplementary material for: ID4-dependent secretion of VEGFA enhances the invasion capability of breast cancer cells and activates YAP/TAZ via integrin β3-VEGFR2 interaction
Source: Cell Death Dis. 2024 Feb 6;15(2):113. doi: 10.1038/s41419-024-06491-2 (PMC10847507; doi:10.1038/s41419-024-06491-2)
Supplement: Supplementary file 4 — Supplementary Figure 3 [file 41419_2024_6491_MOESM4_ESM.pdf]

Supplementary figure 3

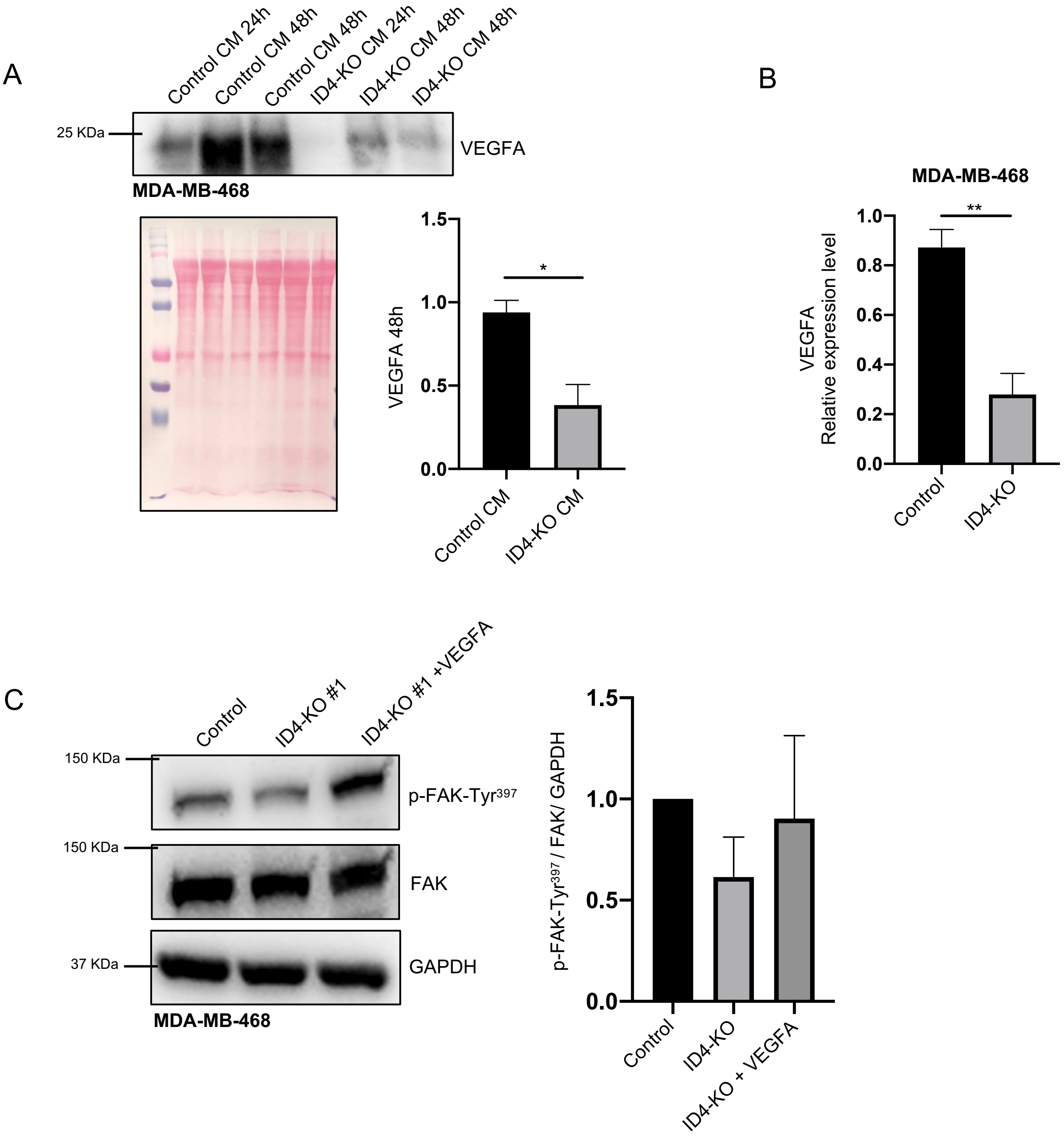

Supplementary Figure 3. A: western blot analysis of VEGFA in CM from MDA-MB-468 Control and ID4-KO cells at 24 and 48h of culture, with quantification graph at 48h. Ponceau S was used for protein normalization. B: real-time PCR analysis of VEGFA in MDA-MB-468 Control and ID4-KO cells. C: western blot analysis of p-FAK and FAK in MDA-MB-468 Control, ID4-KO, and ID4-KO cells treated with recombinant VEGFA for 24h. Data are presented as mean ± SD. *P < 0.05, **P < 0.01, ***P < 0.001, **** P < 0.0001 calculated by Student's t-test (A and B) or One-way Anova (C) on n= 3 experiments.
