# Supplementary figures and images for: ID4-dependent secretion of VEGFA enhances the invasion capability of breast cancer cells and activates YAP/TAZ via integrin β3-VEGFR2 interaction
Source: Cell Death Dis. 2024 Feb 6;15(2):113. doi: 10.1038/s41419-024-06491-2 (PMC10847507; doi:10.1038/s41419-024-06491-2)

# Supplementary figure 4

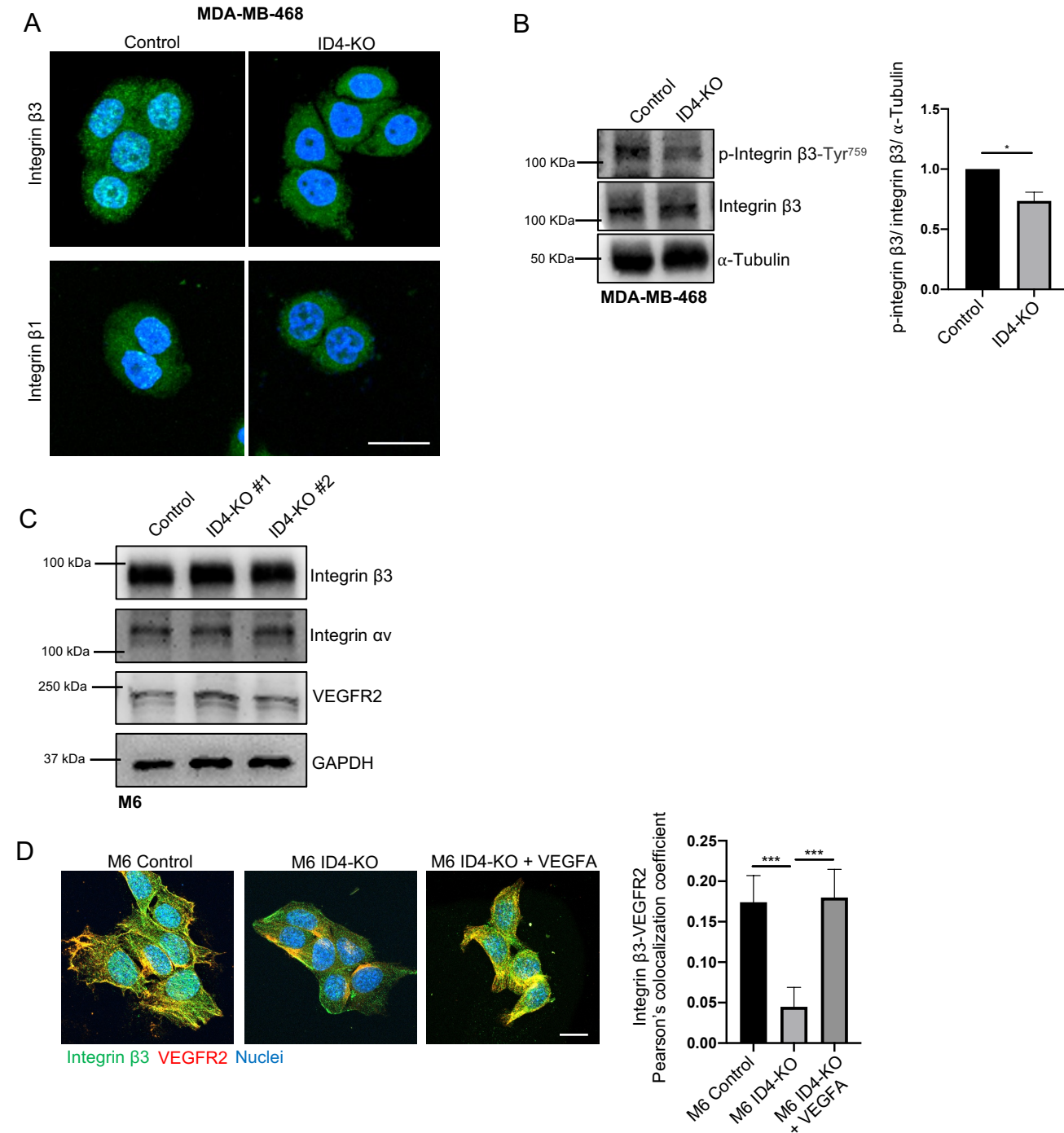

Supplement: Supplementary file 5 — Supplementary Figure 4 [file 41419_2024_6491_MOESM5_ESM.pdf]
