# Supplementary material for: ID4-dependent secretion of VEGFA enhances the invasion capability of breast cancer cells and activates YAP/TAZ via integrin β3-VEGFR2 interaction
Source: Cell Death Dis. 2024 Feb 6;15(2):113. doi: 10.1038/s41419-024-06491-2 (PMC10847507; doi:10.1038/s41419-024-06491-2)
Supplement: Supplementary file 6 — Supplementary Figure 5 [file 41419_2024_6491_MOESM6_ESM.pdf]

Supplementary figure 5

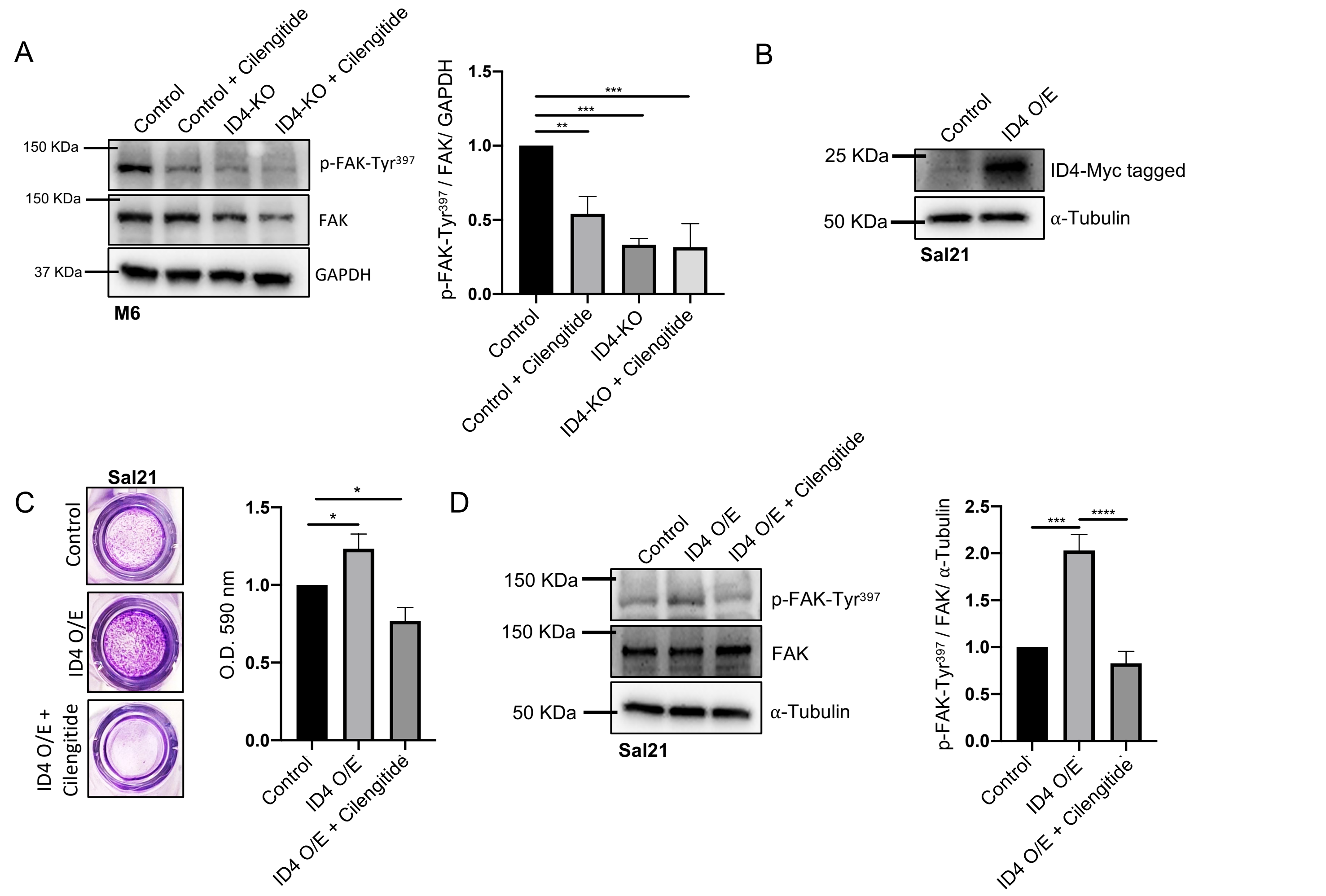

Supplementary figure 5. A: western blot analysis of p-FAK and FAK in M6 Control an ID4-KO cells, with or without Cilengitide treatment, with the relative quantification graph. B: Western Blot analysis of ID4 in Sal21 Control and ID4 O/E cells. C: trasmigration experiment performed with Sal21 Control, ID4 O/E, and ID4 O/E cells treated with Cilengitide. D: Western Blot analysis of p-FAK and FAK in Sal21 Control, ID4 O/E, and ID4 O/E cells treated with Cilengitide, with the relative quantification graph. Data are presented as mean ± SD. *P < 0.05, **P < 0.01, ***P < 0.001, **** P < 0.0001 calculated by One-way Anova test on n=3 experiments.
