# Supplementary material for: ID4-dependent secretion of VEGFA enhances the invasion capability of breast cancer cells and activates YAP/TAZ via integrin β3-VEGFR2 interaction
Source: Cell Death Dis. 2024 Feb 6;15(2):113. doi: 10.1038/s41419-024-06491-2 (PMC10847507; doi:10.1038/s41419-024-06491-2)
Supplement: Supplementary file 7 — Supplementary Figure 6 [file 41419_2024_6491_MOESM7_ESM.pdf]

Supplementary figure 6

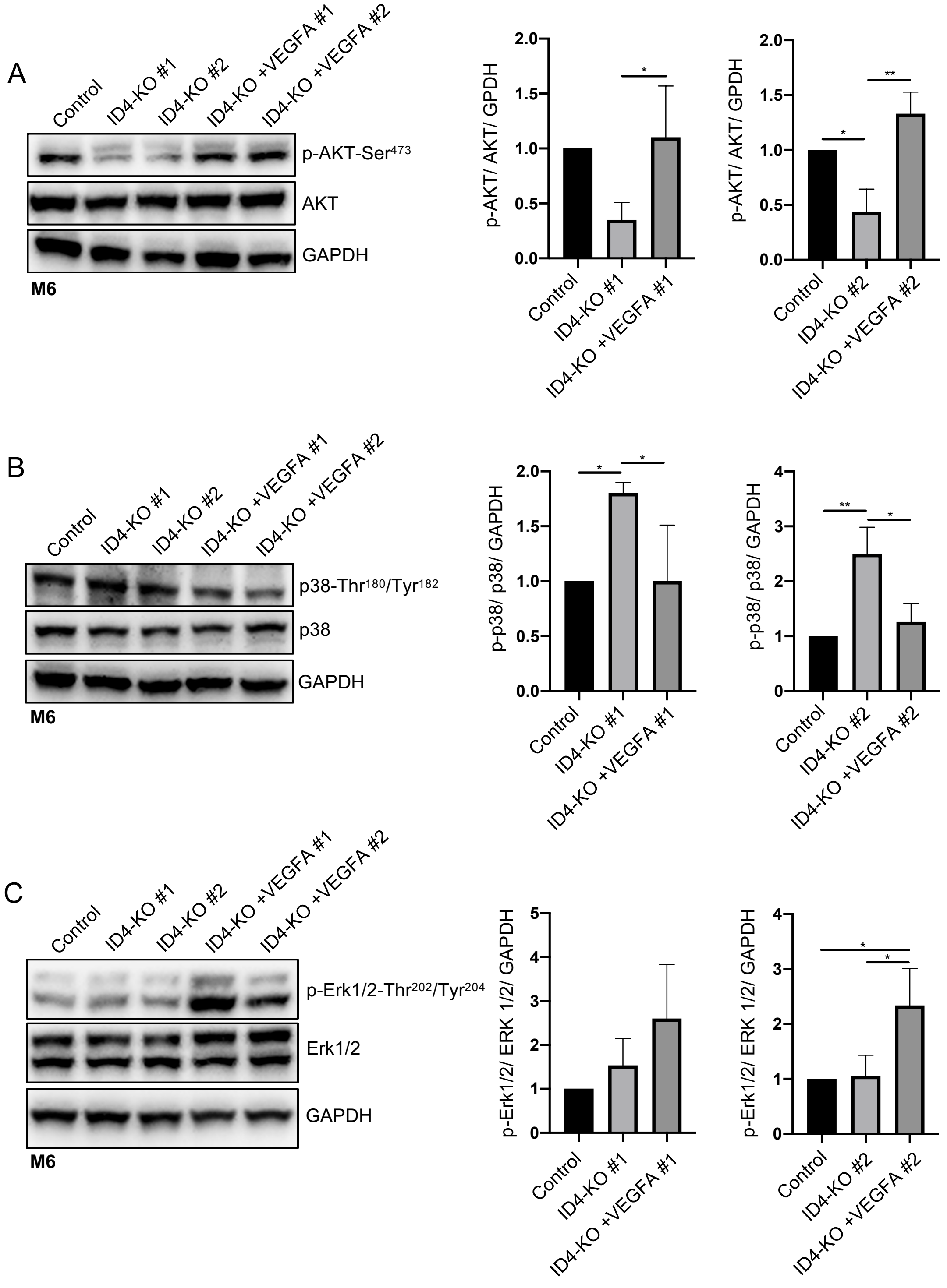

Supplementary figure 6. A: western blot analysis of p-AKT and AKT in M6 Control, ID4-KO, and ID4-KO cells treated with VEGFA for 24h, with the relative quantification graphs. B: western blot analysis of p-p38 and p38 in M6 Control, ID4-KO, and ID4-KO cells treated with VEGFA for 24h, with the relative quantification graphs. C: western blot analysis of p-ERK 1/2 and ERK 1/2 in M6 Control, ID4-KO, and ID4-KO cells treated with VEGFA for 24h, with the relative quantification graphs. Data are presented as mean ± SD. $^{*}P < 0.05$, $^{**}P < 0.01$ calculated by One-way Anova on n= 3 experiments.
