# Supplementary material for: ID4-dependent secretion of VEGFA enhances the invasion capability of breast cancer cells and activates YAP/TAZ via integrin β3-VEGFR2 interaction
Source: Cell Death Dis. 2024 Feb 6;15(2):113. doi: 10.1038/s41419-024-06491-2 (PMC10847507; doi:10.1038/s41419-024-06491-2)
Supplement: Supplementary file 8 — Supplementary Figure 7 [file 41419_2024_6491_MOESM8_ESM.pdf]

Supplementary figure 7

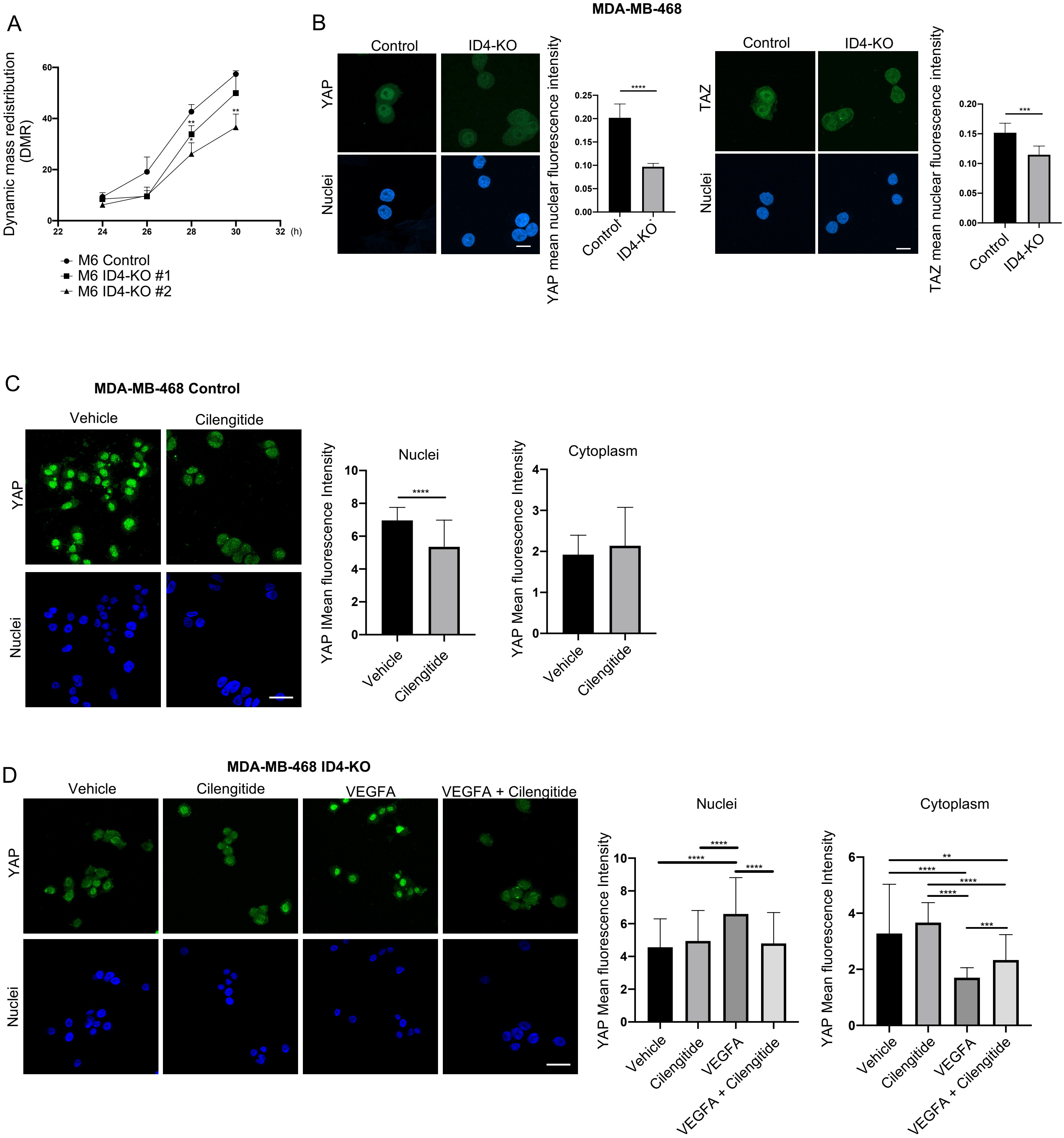

Supplementary figure 7. A: dynamic mass redistribution (DMR) label-free assay in M6 Control and ID4-KO cells at different time points in culture. B: evaluation of YAP (left) and TAZ (right) nuclear staining assessed by immunofluorescence in MDA-MB-468 Control and ID4-KO cells. C: immunofluorescence images of YAP in MDA-MB-468 Control cells treated with vehicle or Cilengitide, and the relative mean fluorescence intensity quantification graph in nuclei and cytoplasm. D: immunofluorescence images of MDA-MB-468 ID4-KO cells treated with vehicle, Cilengitide, and/or VEGFA, and the relative mean fluorescence intensity quantification graph in nuclei and cytoplasm. Scale bar: 20 μm. Data are presented as mean ± SD. *P < 0.05, **P < 0.01, ***P < 0.001, **** P < 0.0001 calculated by Student's t-test (B and C) or One-way Anova test (A and D) on n=3 experiments.
