# Supplementary material for: ID4-dependent secretion of VEGFA enhances the invasion capability of breast cancer cells and activates YAP/TAZ via integrin β3-VEGFR2 interaction
Source: Cell Death Dis. 2024 Feb 6;15(2):113. doi: 10.1038/s41419-024-06491-2 (PMC10847507; doi:10.1038/s41419-024-06491-2)
Supplement: Supplementary file 9 — Supplementary Figure 8 [file 41419_2024_6491_MOESM9_ESM.pdf]

Supplementary figure 8

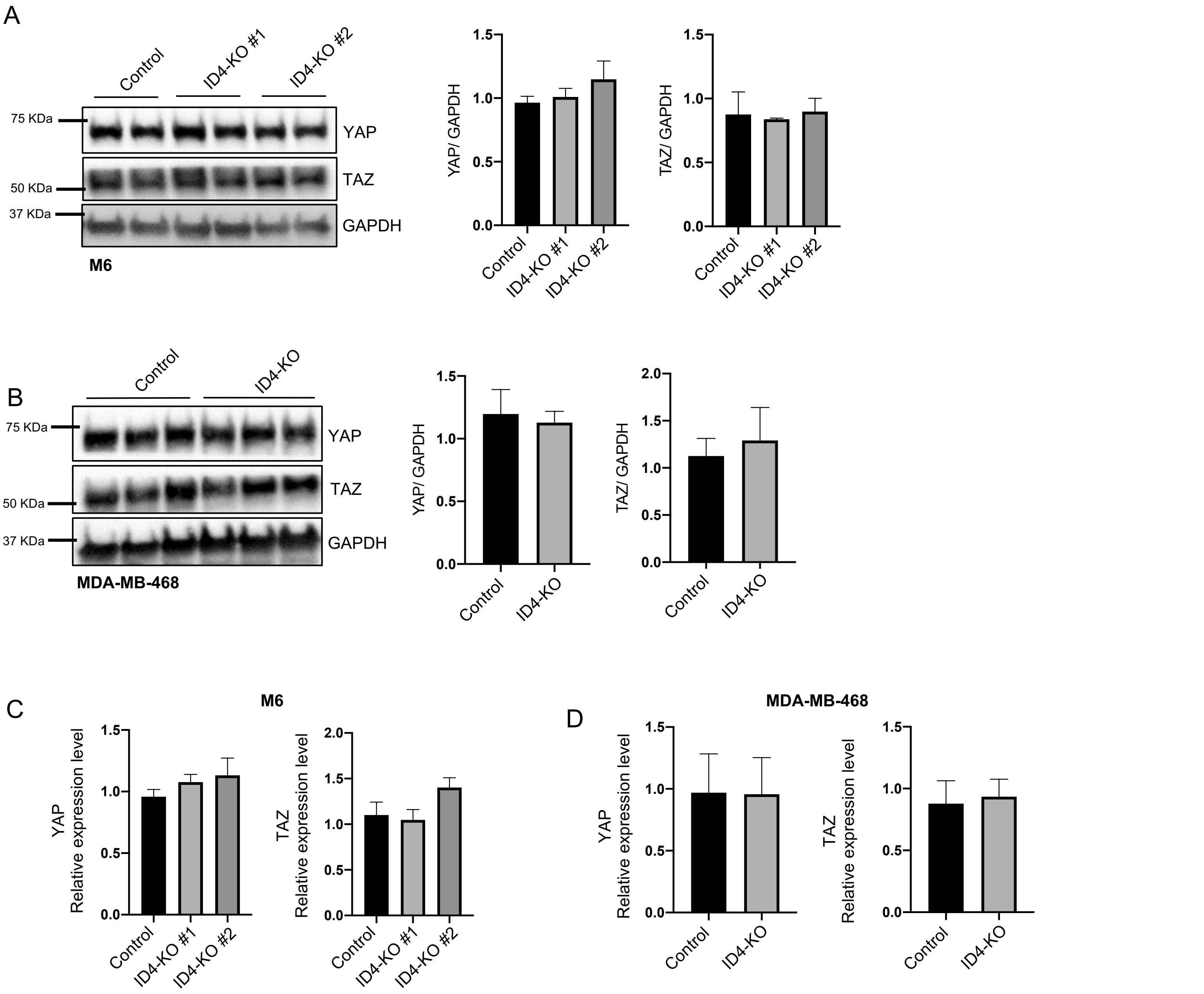

Supplementary figure 8. A: western blot analysis of YAP and TAZ in M6 Control and ID4-KO cells, with the relative quantification graphs. B: western blot analysis of YAP and TAZ in MDA-MB-468 Control and ID4-KO cells, with the relative quantification graphs. C: real-time PCR analysis of YAP and TAZ in M6 Control and ID4-KO cells. D: real-time PCR analysis of YAP and TAZ in MDA-MB-468 Control and ID4-KO cells, n= 3 experiments.
